# Enuresis and upper airway obstruction: BNP and ADH hormones behavior before and after airway surgery

**DOI:** 10.1590/S1677-5538.IBJU.2022.0313

**Published:** 2022-09-20

**Authors:** André Ribeiro, José Murillo Bastos, André Avarese de Figueiredo, Tarssius Capelo Cândido, Wilson Benini Guércio, Bruno O. Zica

**Affiliations:** 1 Universidade Federal de Juiz de Fora Divisão de Otorrinolaringologia Departamento de Cirurgia Juiz de Fora MG Brasil Divisão de Otorrinolaringologia, Departamento de Cirurgia, Universidade Federal de Juiz de Fora (UFJF), Juiz de Fora, MG, Brasil; 2 Universidade Federal de Juiz de Fora Divisão de Urologia Departamento de Cirurgia Juiz de Fora MG Brasil Divisão de Urologia, Departamento de Cirurgia, Universidade Federal de Juiz de Fora (UFJF), Juiz de Fora, MG, Brasil; 3 Hospital e Maternidade Therezinha de Jesus da Faculdade de Ciências Médicas e da Saúde de Juiz de Fora Divisão de Urologia Departamento de Cirurgia Juiz de Fora MG Brasil Divisão de Urologia, Departamento de Cirurgia, Hospital e Maternidade Therezinha de Jesus da Faculdade de Ciências Médicas e da Saúde de Juiz de Fora (HMTJ/Suprema) Juiz de Fora, MG, Brasil

**Keywords:** Nocturnal Enuresis, Natriuretic Peptide, Brain, Urination Disorders

## Abstract

**Introduction:**

Upper airway obstruction (UAO) is a common condition in all pediatric population, with a 27% prevalence. Primary monosymptomatic nocturnal enuresis (PMNE) is a condition related to UAO in 8% to 47% of these children. The specific pathophysiological mechanism of this bond is not well understood. Some authors suggest a connection between brain natrituretic peptide (BNP) and anti-diuretic hormone (ADH) during sleep. The aim of this study was to evaluate hormone profile (ADH and BNP) and improvement in dry nights in a sample of children before and after surgical treatment of the UAO.

**Methods:**

This is a longitudinal prospective interventionist study in children, 5 to 14 years of age, with UAO and PMNE recruited in a specialty outpatient clinic. Children presenting UAO and PMNE were evaluated with a 30-day dry night diary and blood samples were collected to evaluate ADH and BNP before and after upper airway surgery. Data were analyzed prior to surgery and 90-120 days after surgery.

**Results:**

Twenty-one children with a mean age of 9.7 years were included. Mean BNP before surgery was 116.5 ± 126.5 pg/mL and 156.2 ± 112.3 pg/mL after surgery (p<0.01). Mean ADH was 5.8 ± 3.2 pg/mL and 14.6 ± 35.4 before and after surgery, respectively (p=0.26). The percentage of dry nights went from 32.3 ± 24.7 before surgery to 75.4 ± 33.4 after surgery (p<0.01).

**Conclusion:**

Surgery for airway obstruction contributed to an increase in BNP without increasing ADH. A total of 85.8% of the children presented partial or complete improvement of their enuresis.

## INTRODUCTION

Upper airway obstruction (UAO) is a common condition affecting 27% of the pediatric population (1). It is related to anatomical nasal and/or pharyngeal alterations, being adenotonsilar hypertrophy the most incident cause (2).

Primary monosymptomatic nocturnal enuresis (PMNE), which is defined by an intermittent urinary incontinence that occurs during sleep in children from the age of 5 years or more (3), can be related to UAO in an estimated prevalence of 8 to 47% (4). In these cases, surgery to treat upper airway obstruction is associated with a complete PMNE resolution in 31 to 76%, in a follow-up period of 30 to 90 days after surgery (4).

The exact mechanism that controls this improvement remains unknown. The main hypothesis is that UAO causes an increase in negative intrathoracic pressure that causes atrial distension, which will lead to an increase in atrial natriuretic peptide (ANP) release; this would increase the brain natriuretic peptide (BNP) secretion, which induces a release of water and sodium excretion, inhibiting secretion of anti-diuretic hormone (ADH) (4), leading to an increase of nocturnal polyuria.

Some prospective studies have been published (4–7) evaluating UAO and PMNE, but in all of them the criteria to describe upper airway obstruction were not clear, which may have impacted in their results.

We hypothesized that after treating upper obstruction in those children, intrathoracic negative pressure would decrease as so as atrial distention and BNP secretion. In consequence, the water-sodium-ADH secretion mechanism would normalize leading to improvement in enuresis.

The aim of this study was to evaluate the hormone profile (ADH and BNP) and improvement in dry nights in a sample of enuretic children presenting moderate to severe upper airway obstruction before and after surgical treatment of upper airway obstruction.

## MATERIALS AND METHODS

This is a longitudinal prospective interventionist study in a sample of 21 children. The study was approved by the hospital ethics committee (resolution number 2.630.758) and all parents and children signed a free an informed consent and ascent form, respectively. The study was registered in clinical trial (RBR-5pwcs47).

We included children from 5 to 14 years of age with UAO and PMNE that were recruited voluntarily in a tertiary outpatient clinic from May 2018 to November 2020, and whose parents agreed to participate. To confirm that enuresis was monosymptomatic, all children answered a structured questionnaire and fulfilled a voiding diary. Children who presented neurological, psychiatric, metabolic or kidneys illness and presented non monosymptomatic and/or secondary enuresis were not included.

Children presenting UAO and PMNE were evaluated with a 30-day dry night diary and blood samples were collected to evaluate ADH and BNP prior and after surgery of the upper airway. Blood samples were collected early in the morning, about 90 minutes after the child was awaken and a 10 to 12 hours of fasting. All blood samples were collected until 7:30 AM. If, for any reason, children arrived in the laboratory after this time, parents would be instructed to return another day. After arriving in laboratory, the child remained lying down for thirty minutes prior to collecting the blood samples. For both BNP and ADH, blood sample was collected from a peripheral venous puncture. For BNP, 0.8 mL of blood was centrifuged for 10 minutes with 2,200 grams of gravity in 18 Celsius degrees; after separation in serum, clot and gel, serum was submitted to eletrochemiluminescence technique. For ADH, 2.5 mL of blood was separated in a test tube with EDTA - *Ethylenediamine tetraacetic acid* and was centrifuged for 10 minutes in 3,000 rpm and then, frozen to minus 20 Celsius degree. ADH was dosed from frozen plasma by radioimmunoassay technique.

After completing the evaluation, upper airway surgery was scheduled and was done by only one surgeon. The surgery was performed under general anesthesia and oral intubation.

All data were analyzed prior to airway surgery and 90-120 days after it.

Children were not submitted to any other kind of treatment for enuresis, as urotherapy, alarm or medication.

**Figure 1:**
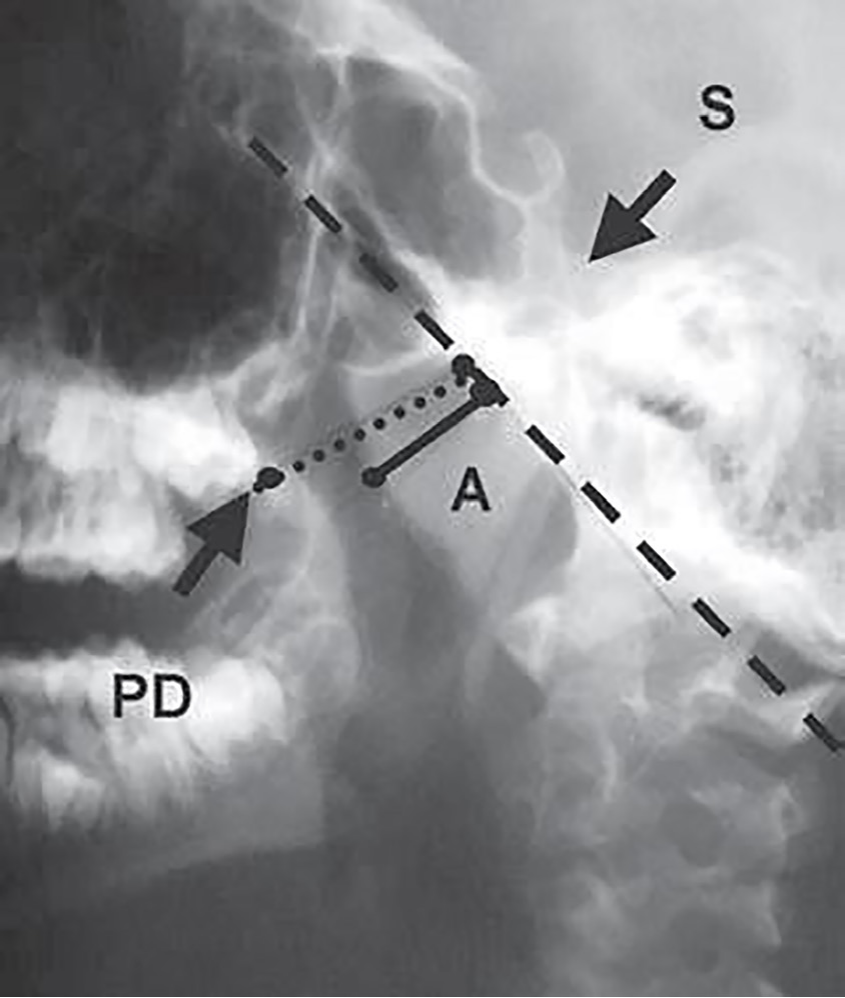
Cavum X-Ray - adenoid nasopharynx ratio (ANR) – black line is the measure of the adenoid / dotted line is nasopharynx measure; ANR is the result of the division of the measures of the black line by the dotted line.

**Figure 2:**
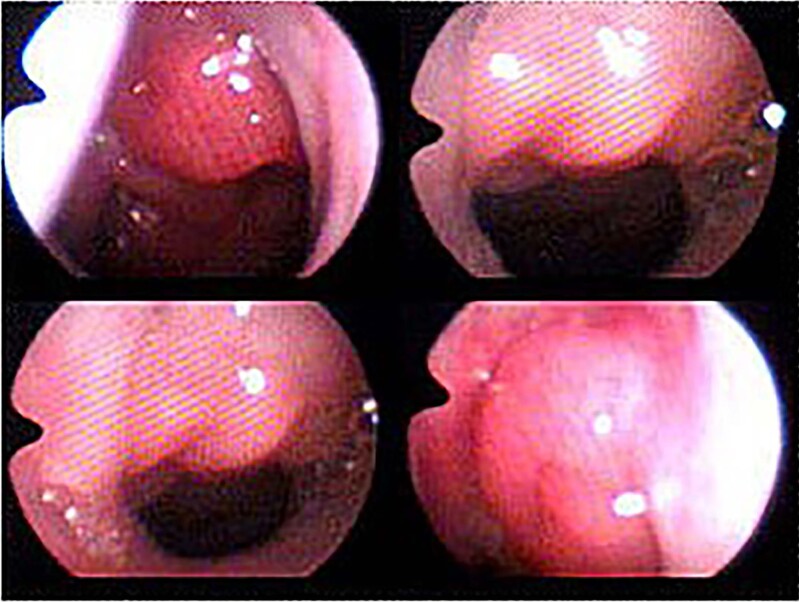
Choana nasal endoscopic view – Grades I (up to 25% of choana obstruction), II (25% to 50% of choana obstruction), III (50% to 75% of choana obstruction) and IV (more than 75% of choana obstruction) - from left to right and from top to bottom.

Upper airway obstruction was objectively defined using cavum x-ray and nasal endoscopy. The criteria used was adenoid nasopharynx ratio (ANR) greater than 0.66 in the X-ray (8) and Brodsky 3 or 4 for nasal endoscopy (9) (Figures 1 and 2).

The Apnea / Hypopnea Index (AHI) is a way of classifying the degree of sleeping apnea. In children, up to 5 events per hour is classified as mild apnea, from 5 to 15 as moderate and severe if more than 15 events per hour (10).

To predict the Apnea / Hypopnea Index (AHI) we used the formula of Klijajic Z et al. AHI = 3.2 x modified Mallampati score + 1.13 tonsillar size – 1.65 (10).

Sample calculation was made based on Kovasevic et al. study (4) using www.biomath.info/power/ttest.htm (11) for a 80% power of study and an Alfa of 0.05.

For statistical analysis we used JASP free platform (12). For assumption checks we used Shapiro-Wilk test. Parametric data was analyzed using t-test and for non-parametric data we used Wilcoxon test. Central trend and dispersion measures were used for the analysis. Significance was considered when p-value was ≤ 0.05.

## RESULTS

**Figure 3:**
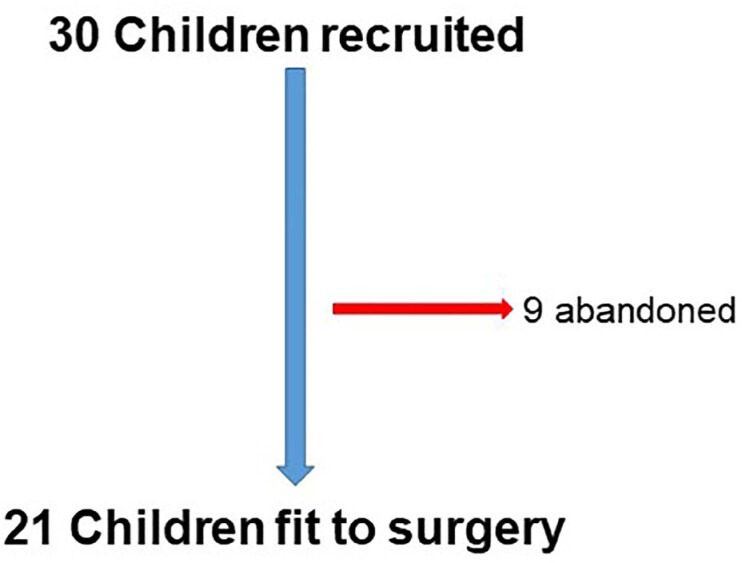
Patient recruitment flowchart.

A total of 30 children presenting PMNE and UAO that filled inclusion criteria were recruited. Of those, 9 discontinued follow-up due to fear of surgery and it's possible complications and also claiming difficulty to continue follow-up after surgery. These patients received other clinical treatments for UAO and PMNE. Therefore, a total of 21 children, 6 to 14 (9.7 ± 2.49) years of age, being 13 boys, have completed all the protocol (hormones tests and dry night diary before and after surgery) (Figure-3).

After completing all the protocol an increase in BNP levels (p=0.001) and in the number of dry nights (p=0.001) were found. No difference was found for ADH levels (p=0.26) as demonstrated in Table-1.

**Table 1:** Mean variation from before and 90 to 120 days after airway surgery.

	Before surgery	After surgery	p value
BNP (pg/mL)	116.5 ± 126.5	156.2 ± 112.3	< 0.01
ADH (pg/mL)	5.8 ± 3.2	14.6 ± 35.4	0.26
Dry nights %	32.3 ± 24.7	75.4 ± 33.4	< 0.01

According to the International Children's Continence Society criteria (ICCS), of the 21 children, 9 presented total improvement of enuresis, 9 presented partial improvement, and 3 did not present any improvement (Table-2).

**Table 2:** Improvement of Enuresis based on the International Children's Continence Society (ICCS) criteria.

ICCS Criteria		Absolute Frequency	Relative Frequency
	< 50% of improvement	3	14.2%
50% - 99% of improvement		9	42.9%
100% of improvement		9	42.9%

## DISCUSSION

The present study is one of the few that have evaluated BNP and ADH profile in children presenting PMNE and UAO. The results presented herein demonstrated an increase in BNP levels and no difference in ADH levels after correction of UAO in those children. Considering the ICCS criteria (13, 14) it was observed an 85.8% improvement in enuresis, being half of those (42.9%) a complete response. These findings show an improvement better than 68% described by Lehmann et al. in their systematic review (15).

Perhaps the increase of BNP levels has a relationship with the blood collection time, as we will describe further. The ideal time for blood collection is exactly the moment child is urinating, during the night; but it is virtually impossible for logistical reasons.

To avoid selection and analysis bias, only PMNE children were included in the present study.

Some studies suggests that upper airway obstruction causes negative intrathoracic pressure, leading to cardiac wall distension, which results in the release of atrial natriuretic peptide – ANP which induces BNP secretion (16). These hormones cause increase in water and sodium excretion, which inhibit vasopressin secretion. Therefore, it was hypothesized that releasing upper airway could normalize serum levels of these hormones (BNP would fall and ADH would increase), leading to improvement of enuresis. This is what was proposed by some studies (4–7). Differently from those findings, we observed an increase in BNP levels and no changes in ADH after airway surgery; although our ADH mean increases almost three times, we have some outliers that possibly interfered in ours results. But even if we analyze data excluding outliers, we would find the following results of ADH hormone: mean before surgery 4.59 ± 1.83 and 17.03 ± 39.18 after surgery (p 0.19). These differences in our findings may also be related to a higher mean age or the moderate to severe upper airway obstruction of our series.

As we present different results than what we expected, related to BNP and ADH, we have to infer that hormonal mediation of PMNE was not valid in our sample. We expected a decrease in BNP and an increase in ADH, both with statistical significance.

The mean age of our sample is a bit higher than others prospective studies (4–7) which may have influenced our results, since it has been shown that 15% of enuretic children improves their symptoms every year regardless of any treatment. Another factor that can be associated with our better results with the UAO surgery is the degree of obstruction in our patients. The mean ANR found our sample was 0.78 (0.68-0.88), which means that the children had a moderate to severe obstruction, other than that, we used the formula of Klijajic Z. et al. (10) to predict the Apnea / Hypopnea Index (AHI) (AHI = 3.2 x modified Mallampati score + 1.13 tonsillar size – 1.65); in our sample the average of this estimated index was 7.8, which is considered moderate. A greater degree of obstruction may have a greater impact on the cascade of events related to enuresis in this population and also a greater impact on their sleep quality.

Enuretic children are considered to be *deep sleepers* (17). Children presenting UAO are oral breathers which makes them hyperexcited. This hyper excitation is related to a non-physiological breathing mechanism during sleep, caused by UAO, which increases muscular effort and norepinephrine levels. This condition causes a constant stimulus increasing their excitation threshold and making it difficult for them to wake up. In order to catch up deep sleep phases, as REM (rapid eyes movement), these children would develop inhibition afference mechanisms to effectively rest at night, other than that, if there is a constant arousal stimulus (from the airways) the arousal thresholds will increase in order to preserve sleep. The problem is, these children will keep high norepinephrine levels during the day (because of a bad sleep night that will prevent them to sleep well next night), which will keep them with high excitation threshold, consequently increasing afference inhibition, perpetuating this vicious cycle.

Besides that, recently Sun et al. (18) proposed that the winter season and high severity of initial symptoms are two high risk factors for desmopressin treatment failure; this is corroborated by Bastos Netto and Bessa Junior (19) in their editorial comment paper; they also describe some differences in sleeping architecture which could cause influence in this multifactorial and very complex mechanism of nocturnal urinary production. Just another piece of this complex puzzle.

Our follow up (90 to 120 days) was longer than Kovasevic et al. study - 30 days - (4, 5), but shorter than Fakhim et al. (7) - 180 days. In our point of view, this extended follow up allowed a better adaptation of circadian cycle of patients, contributing for an 85.8% improvement of enuresis.

So, the results presented herein are applicable to those children with more severe upper airway obstruction. This demonstrates that in a group of enuretic children with severe upper airway obstruction, surgery to release airway has to be tightly considered as a therapeutic option.

Although our sample is small, it exceeds the sample calculation for a clinical trial with a comparative group (14 patients in each group), which is being constituted in continuity of our research; this part of the major study already shows how airway surgery is an effective treatment for these specific enuretic children. The pathophysiology of enuresis in children with UAO remains unknown. Comparative studies, especially prospective ones are necessary to check the role of hormones in the control of enuresis for what we can definitively include or exclude BNP and ADH from this chain of events.

Therefore, the ideal study design would be one in which we could collect blood samples during night at the exact moment the child was urinating but unfortunately, we could not do so but only after the child had wakened; eventually this could explain the absence of changing in ADH measures. Although our sample size was within the sample previously calculated we believe that a large number of children could demonstrate different results, especially regarding ADH, which was almost three times greater after surgery and no significant difference was found; other than that, as we focused on the hormones behavior, we didn't consider nocturnal urine production as a variable; this can be considered another weakness of our research. To better understand these differences, future studies are needed, including a comparative group.

## CONCLUSIONS

Evaluation of hormones profile in children presenting PMNE and upper airway obstruction showed high levels of BNP and low levels of ADH before airway surgical treatment. After that, there is an increase in both BNP and ADH levels, although mean variation of ADH was not statistically significant.

Clinically, upper airway surgery was able to improve the number of dry nights in 85.7% these children, with complete response in 42.8 %.

Improvement of enuresis after surgery could not be explained by the variations in BNP and ADH.
